# Broad‐range metalloprotease profiling in plants uncovers immunity provided by defence‐related metalloenzyme

**DOI:** 10.1111/nph.18200

**Published:** 2022-05-26

**Authors:** Kyoko Morimoto, Daniel Krahn, Farnusch Kaschani, Digby Hopkinson‐Woolley, Anna Gee, Pierre Buscaill, Shabaz Mohammed, Stephan A. Sieber, Benjamin F. Cravatt, Christopher J. Schofield, Renier A. L. van der Hoorn

**Affiliations:** ^1^ The Plant Chemetics Laboratory Department of Plant Sciences University of Oxford South Parks Road Oxford OX1 3RB UK; ^2^ Department of Chemistry and the Ineos Oxford Institute for Antimcrobial Research University of Oxford Mansfield Road Oxford OX1 3TA UK; ^3^ The Plant Chemetics Laboratory Max Planck Institute for Plant Breeding Research Cologne 50829 Germany; ^4^ Department of Biochemistry University of Oxford Oxford OX1 3QU UK; ^5^ Department of Chemistry The Skaggs Institute for Chemical Biology The Scripps Research Institute La Jolla CA 92037 USA

**Keywords:** Arabidopsis, immunity, metalloproteases, photoaffinity labelling, PRp27

## Abstract

- Plants encode > 100 metalloproteases representing > 19 different protein families. Tools to study this large and diverse class of proteases have not yet been introduced into plant research.
- We describe the use of hydroxamate‐based photoaffinity probes to explore plant proteomes for metalloproteases.
- We detected labelling of 23 metalloproteases in leaf extracts of the model plant *Arabidopsis thaliana* that belong to nine different metalloprotease families and localize to different subcellular compartments. The probes identified several chloroplastic FtsH proteases, vacuolar aspartyl aminopeptidase DAP1, peroxisomal metalloprotease PMX16, extracellular matrix metalloproteases and many cytosolic metalloproteases. We also identified nonproteolytic metallohydrolases involved in the release of auxin and in the urea cycle. Studies on tobacco plants (*Nicotiana benthamiana*) infected with the bacterial plant pathogen *Pseudomonas syringae* uncovered the induced labelling of PRp27, a secreted protein with implicated metalloprotease activity. PRp27 overexpression increases resistance, and PRp27 mutants lacking metal binding site are no longer labelled, but still show increased immunity.
- Collectively, these studies reveal the power of broad‐range metalloprotease profiling in plants using hydroxamate‐based probes.

## Introduction

Metalloproteases fulfil diverse and important functions in plants. All metalloproteases use a metal ion to polarize the amide carbonyl in the peptide bond and use an activated water molecule for nucleophilic attack (Van der Hoorn, 2008). The metal ion is coordinated in the active site via His, Asp and Glu residues.

The Arabidopsis genome includes > 130 genes encoding metalloproteases and close homologues. The MEROPS peptidase database (Rawlings *et al*., 2018) classifies these metalloproteases into 19 families, based on sufficient sequence similarity to the peptidase type member. These 19 families are grouped into nine clans based on structural similarities, suggesting a common evolutionary origin.

Clan MA contains 48 proteases in nine families that all bind a single zinc active site ion via an HExxH motif. Proteases in family M50 (clan MM) also bind a zinc ion via an HExxH motif, but these are more ancient and are thought to have evolved independently (Kinch *et al*., 2006). A ‘reverse’ motif (HxxE(H)) to bind metal ions also appears to have evolved twice independently. The 14 proteases of family M16 (clan ME) bind zinc ions via an HxxEH motif, and the four proteases of family M14 (clan MC) bind metal ions via an HxxE motif. Clan MH contains 22 proteases in three families that bind two zinc ions with HxD and EE motifs. There are five proteases in family M38 (clan MJ) and 14 proteases in family M67 (clan MP) that bind nickel and zinc ions with the HxH motif. Finally, there are four proteases in family M17 (clan MF) that carry the metal‐binding DxExR motif, and 13 proteases in family M24 (clan MG) that bind cobalt and magnesium ions with scattered residues.

All these proteases bind their metal ion within a peptide binding groove that can recognize specific peptide sequences and structures. Several metalloproteases act as exopeptidases to remove targeting peptides, such as site‐2‐peptidase, S2P (M50; Kinch *et al*., 2006). In many metalloproteases, the peptide‐binding groove is obstructed from one or the other side, restricting their activity to terminal residues (exopeptidases), either N‐terminally (aminopeptidases) or C‐terminally (carboxypeptidases). For instance, methionine aminopeptidases (MAPs, family M24) remove the initiator Met residues from nascent proteins (Liu *et al*., 1998). Some of the exopeptidases cleave after two residues instead of one, making them aminodipeptidases and carboxydipeptidases, respectively. Peptide bonds are amides and homologues of metalloproteases are able to cleave amide bonds in other molecules and play roles in primary or secondary metabolism, examples including urease (M38; Witte *et al*., 2005) and the M20 hydrolases that cleave the amide bond to release auxin from amino acid conjugates (LeClere *et al*., 2002).

Metalloproteases act at virtually every region of a plant cell: they are found in the nucleus, cytoplasm, vacuole, peroxisomes, mitochondria and chloroplasts. Additionally, although many are soluble enzymes, others are embedded in membranes via transmembrane domains (e.g. FtsHs) or glycosylphosphatidylinositol anchors (e.g. matrix metalloproteases (MMPs)). These membrane‐localized metalloproteases act on substrates on either side of the membrane (e.g. stroma for FtsH and apoplast for MMPs), but some, such as S2P, are intramembrane proteases, cleaving transmembrane proteins within the membrane.

Metalloproteases can be inhibited by nonspecific metal ion chelators such as EDTA and phenanthroline, which can extract the metal ion from the enzymes, and by hydroxamate‐based inhibitors, which bind directly to the metal ion in the enzyme and block the substrate binding groove. Examples of these noncovalently binding hydroxamate inhibitors are marimastat and batimastat, which are used to treat cancer (Rasmussen & McCann, 1997). Some covalent inhibitors exist for specific metalloprotease families, such as fumagillin for MAPs (Liu *et al*., 1998), but covalent inhibitors are not known for most metalloproteases.

Because metalloproteases irreversibly determine the fate of proteins, their activation and activity are tightly regulated. Activation is usually caused by the sequential removal of autoinhibitory prodomains. After prodomain removal, these proteases are regulated with posttranslational modifications and inhibitors. The activity of metalloproteases is therefore difficult to predict based on transcript and protein levels.

Protease activity profiling is a powerful functional proteomics approach to detect the active state of proteases using activity‐based probes that label protease active states. These probes mimic protease substrates, but contain a reactive group that locks the catalytic mechanism in the covalent intermediate state. Examples of these activity‐based probes validated on plants are DCG‐04 for papain‐like Cys proteases (Van der Hoorn *et al*., 2004), AMS101 for vacuolar processing enzymes (Misas‐Villamil *et al*., 2013), MV151 for the proteasome (Gu *et al*., 2010), fluorophosphonates (FPs) for Ser hydrolases (Kaschani *et al*., 2009a) and JJB70 for glycosidases (Chandrasekar *et al*., 2014).

Metalloproteases, however, are difficult to trap with covalent inhibitors because they do not make covalent intermediates with their substrates, although they are often sensitive to inhibition by noncovalently binding hydroxamate‐based inhibitors. This property inspired Cravatt *et al*. to generate hydroxamate‐based probes that contain an alkyne minitag and a photoreactive benzophenone group to create covalent bonds with the target protease upon ultraviolet (UV) irradiation (Saghatelian *et al*., 2004). These hydroxamate‐based probes only label active metalloproteases, not the inactive precursors (zymogens) (Saghatelian *et al*., 2004). These hydroxamate probes have been used to study activation of matrix metalloprotease MMP‐2 upon oxidation of the cysteine switch motif (Keow *et al*., 2012), demonstrating that the zymogen cannot be labelled. Further studies have identified six endogenous metalloproteases from human cancer cells, which represent all major branches of this enzyme superfamily, indicating that hydroxamate probes can offer broad‐range metalloprotease profiling (Sieber *et al*., 2006). Although these probes do not react with the active site in an activity‐dependent manner, the labelling reports the availability of the active site containing a metal ion, and hence indicates that the enzyme is active.

Here, we report studies investigating use of alkyne‐hydroxamate photoaffinity probes to profile metalloproteases in Arabidopsis leaf extracts. Alkyne minitags can be coupled to biotin or fluorophores using ‘click‐chemistry’ (Kaschani *et al*., 2009b). We identified biotin‐labelled proteins by MS and validated interactions by employing fluorescently labelled proteins and mutants. We also investigated differential labelling of metalloproteases in the apoplast of *Nicotiana benthamiana* upon infection with *Pseudomonas syringae*. Together, these data illustrate the power of broad‐range metalloprotease profiling in plants.

## Materials and Methods

### Chemical probes

Cy3, Cy5 and Biotin Picolyl Azides were purchased from Click Chemistry Tools (Scottsdale, AZ, USA). The synthesis of the initial hydroxamate probes has been described previously (Sieber *et al*., 2006). DK‐01 was synthesized as described in the Supporting Information Methods S1, and aliquots are available upon request.

### Plant materials and growth conditions

Arabidopsis T‐DNA insertion lines *prep2* (SALK_133220), *mpa1‐1* (SALK_006826) and *mpa1‐2* (SALK_049838C) were obtained from the Nottingham Arabidopsis Stock Centre (NASC; Nottingham, UK). Mutants *prep1/2* (Cederholm *et al*., 2009); *apm1‐1*(−/+) and *apm1‐1*(−/−) complemented with IRAP (Hosein *et al*., 2010); and *top1‐3* and *top2‐1* single mutants and the *top1‐3/top2‐1* double mutant (Moreau *et al*., 2013) were described previously. All mutants used were in the Col‐0 background. Arabidopsis plants were grown in soil at 25°C under a 16 h : 8 h, light : dark photoperiod at a photon density of 100 ± 10 µmol photons m^−2^ s^−1^. *Nicotiana benthamiana* plants were grown in a growth chamber at 22°C and *c*. 60% relative humidity with a 16 h photoperiod and a light intensity of 200 ± 50 µmol photons m^−2^ s^−1^.

### Total RNA extraction and cDNA synthesis

Four leaf discs (0.9 cm in diameter) were taken from the leaves infected with *Pto*DC3000 at 2 d postinfection (dpi), frozen in liquid nitrogen and pulverized using a tissue lyser. Total RNA was extracted from leaf powder using Trizol (Thermo Fisher Inc., Waltham, MA, USA) according to the manufacturer’s instructions. Genomic DNA was removed by in‐solution digest with the Qiagen RNAse‐free DNAse kit, followed by RNA purification with the Qiagen RNeasy kit, following the manufacturer’s instructions (Qiagen). RNA concentration was measured using a NanoDrop (Thermo Fisher Inc.) and 500 ng of RNA was used for cDNA synthesis using Oligo(dT)12‐18 primer and SuperScript™ II Reverse Transcriptase (Invitrogen), following the manufacturer’s instructions.

### Molecular cloning

All constructs were generated using Golden Gate (GG) cloning (Engler *et al*., 2014). The primers and (generated) plasmids are listed in Tables S1 and S2, respectively.

The open reading frame (ORF) of *NbPRp27* was amplified with the primers listed in Table S2 from *N. benthamiana* cDNA and assembled with the pJK001c binary vector (Paulus *et al*., 2020), 35S promoter module and 35S terminator module for transient protein expression in *N*. *bentamiana*, resulting in pPB046.

The 300 bp fragment of NbPRp27 (Sequence A) used for virus‐induced gene silencing (VIGS) was amplified with the primers listed in Table S2 from *N. benthamiana* cDNA. The fragment was cloned into binary TRV2 vector (pJK037) using *Bsa*1 restriction sites resulting in binary vector pPB037.

The ORF of *Nb*PRp27 (Sequence B) was amplified with primers 1 + 2 listed in Table S2 from *N. benthamiana* cDNA infected with *Pto*DC3000 and assembled with the pJK001c binary vector (Paulus *et al*., 2020), 35S promoter module and 35S terminator module for transient protein expression in *N. benthamiana*, resulting in pPB046. Substitution mutants were generated by GG cloning of fragments generated by PCR from the pPB046 template using primers 1 + 2 in combination with mutagenesis primers 8 + 9, 7 + 8 and 10 + 11 (Table S2), and assembled with the pJK001c binary vector (Paulus *et al*., 2020), 35S promoter module and 35S terminator module, to produce pKM001(E123Q), pKM002(H122F) and pKM003(H126F), respectively.

For expression of His‐tagged *Nb*PRp27 in *Escherichia coli*, the ORF of *NbPRp27* without the sequence encoding the signal peptide was amplified from pPB046 using primers 5 + 6 (Table S2) and assembled in expression vector pJK082 (Kourelis *et al*., 2020), with pJK122i (His‐tag; Kourelis *et al*., 2020), pJP001 (T7 promoter; Kourelis *et al*., 2020) and pJP002 (T7 terminator; Kourelis *et al*., 2020), resulting in pKM005. Substitution mutants were similarly generated by GG cloning of fragments generated by PCR from pKM002(H122F), pKM001(E123Q) and pKM003(H126F) templates using primers 5 + 6 (Table S2) and cloned into pJK082 as described above, to produce pKM008 (H122F), pKM011(E123Q) and pKM015(H126F), respectively.

### *Arabidopsis* protein extraction

Rosette leaves of 4‐ to 5‐wk‐old *Arabidopsis* plants were ground in a 1.5 ml tube with a pestle. Total proteins were extracted in the extraction buffer (1× PBS, pH 7.4 (8.1 mM Na_2_HPO_4_, 1.47 mM KH_2_PO_4_, 137 mM NaCl, 2.7 mM KCl)), equivalent to five times the fresh weight of the plant tissues. The extracts were centrifuged at 15 000 ***g*** for 20 min at 4°C; the supernatant containing the soluble proteins was retained. The protein concentration of the supernatant was measured using the DC protein assay kit (Bio‐Rad) and adjusted to 2 mg ml^−1^ with extraction buffer. The resulting extracts were used for labelling. For pH course experiments, protein extraction was carried out in various pH buffers instead of 1× PBS: 50 mM sodium acetate (pH 3, 4 and 5), MES (pH 6), MOPS (pH 7 and 8) or Tris (pH 9).

### Agroinfiltration

*Agrobacterium tumefaciens* GV3101 (pMP90) was used for agroinfiltration of *N. benthamiana*. Agrobacteria were grown in Luria‐Bertani (LB) medium with 25 μg ml^−1^ rifampicin and 50 μg ml^−1^ kanamycin at 28°C. For transient expression of proteins in *N. benthamiana*, overnight cultures of *Agrobacterium* carrying binary vectors were harvested by centrifugation. Bacterial cells were resuspended in agroinfiltration buffer (10 mM MgCl_2_, 10 mM MES pH 5.6, 200 μM acetosyringone) at an OD_600_ of 0.5. After 3 h at 28°C, cells were infiltrated into leaves of 4‐wk‐old *N. benthamiana*. The infected plants were grown in a growth chamber until use.

### *P. syringae* infection assays

Wild‐type *P. syringae* pv *tomato* DC3000 (*Pto*DC3000) and the *ΔhrpA* (Roine *et al*., 1997) and *ΔhopQ1‐1* (Wei *et al*., 2007) mutants were used for disease assays. *Pto*DC3000 strains were grown in LB medium with 25 μg ml^−1^ rifampicin at 28°C. *Pto*DC3000 cultures were grown overnight and resuspended in sterile water at an OD_600_ of 0.001. Bacterial cultures were infiltrated into leaves of 4‐ to 5‐wk‐old *N. benthamiana* plants. The leaves were harvested at 2 dpi.

### Production of recombinant His‐tagged PRp27 in *E. coli*

The pET28a‐PRp27 expression vector was transformed into *E. coli* Rosetta (DE3) pLysS cells (Novagen, Madison, WI, USA). The bacterial cultures were grown in liquid LB medium at 37°C to an OD_600_ of 0.5 and isopropyl‐β‐d‐thiogalactoside (IPTG) was added to a final concentration of 0.5 mM. The cultures were incubated overnight at 22°C. Total proteins were extracted in 1× PBS, pH 7.4, containing 1 mg ml^−1^ lysozyme by incubation on ice for 1 h followed by sonication three times for 3 s. The extracts were centrifuged at 15 000 ***g*** for 10 min at 4°C and supernatants containing the soluble proteins were 10‐fold diluted in 1× PBS, pH 7.4, and used for labelling.

### Virus‐induced gene silencing

*Agrobacterium* cultures were grown overnight and resuspended in agroinfiltration buffer at an OD_600_ of 0.5. Bacteria containing pPB037 (*TRV::PRp27*) or *TRV2::GFP* (Liu *et al*., 2002) were mixed 1 : 1 with bacteria containing TRV1 (Liu *et al*., 2002). After incubation for 3 h at room temperature, the mixed cultures were infiltrated into leaves of 14‐d‐old *N. benthamiana* plants. The infiltrated seedlings were grown for 3–4 wk in a growth chamber until use.

### Isolation of apoplastic fluids (AFs)

*Nicotiana benthamiana* leaves were submerged in ice‐cold water and vacuum infiltrated in a 50 ml syringe with a plunger. The surface of water‐infiltrated leaves was dried with absorbing paper and leaves were carefully mounted in an empty 20 ml syringe, which was placed in a 50 ml tube. Apoplastic fluids were collected by centrifugation at 2000 ***g*** at 4°C for 10 min. Apoplastic fluids were buffered with 1 : 20 volume of 1 M sodium acetate, pH 5.6, at the final concentration of 50 mM and immediately used for labelling.

### Labelling of plant extracts

**Fig. 1:**
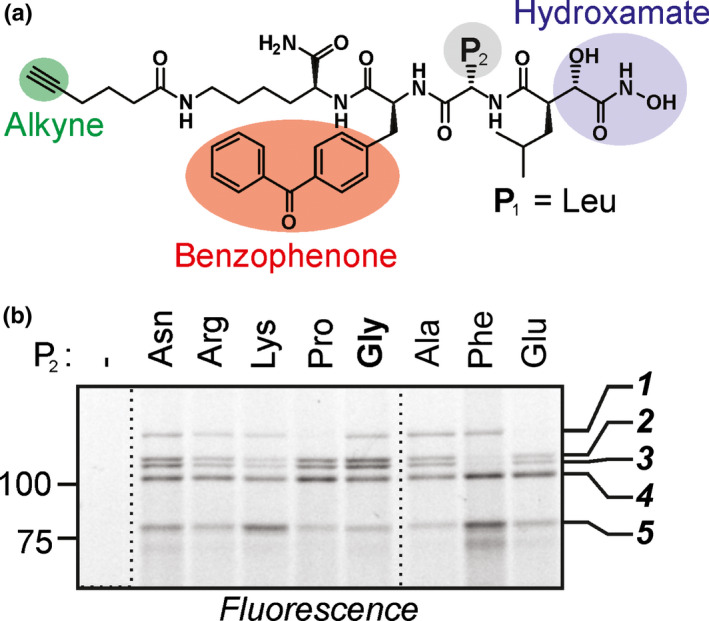
Hydroxamate‐based probes display five polymorphic signals in Arabidopsis leaf extracts. (a) General structure of the used probes. All compounds carry a hydroxamate zinc‐binding group (blue), an alkyne minitag (green) and a photoreactive benzophenone group (red), connected through a dipeptide with various amino acid residues at P_2_ (grey). (b) Differential labelling profiles with different hydroxamate probes. Arabidopsis leaf extracts were labelled with and without 1 µM probes by UV irradiation at 366 nm for 30 min on ice. Alkyne‐labelled proteins were coupled to a fluorophore using click‐chemistry with rhodamine‐azide. Proteins were separated on SDS‐PAGE gels and fluorescent proteins were detected by in‐gel fluorescence scanning (ex488/em520). Five specific signals are indicated on the right.

The procedure used to produce the data presented in Fig. 1 is described in Methods S1. All probes were prepared as stock solutions in dimethyl sulphoxide (DMSO). Equal volumes of DMSO were used as a no‐probe control. Leaf extracts or AFs were mixed with the probes or DMSO in 1.5 ml microcentrifuge tubes. The mixtures were loaded in an ice‐cooled 96‐well microplate in 100–200 μl fractions and irradiated at 245 nm using a UV lamp (ENF 280C; Spectroline, Melville, NY, USA) for 30 min on ice. The labelling reactions were quenched by performing precipitation with four volumes of 100% ice‐cold acetone. The protein pellets were resuspended in 44 μl of PBS‐SDS buffer (1× PBS, pH 7.4, 1% (v/v) SDS), heated at 90°C for 10 min and used for click‐chemistry.

### Click‐chemistry

For click‐chemistry, 2.5 μM Cy3/Cy5 picolyl azide or 25 μM biotin picolyl azide (all from DMSO stocks) and the premixture of 100 μM tris[(1‐benzyl‐1H‐1,2,3‐triazol4‐yl)methyl]amine (TBTA, 3.4 mM stock in DMSO : *tert*‐butanol 1 : 4); 2 mM Tris(2‐carboxyethyl)phosphine hydrochloride (TCEP, 100 mM stock in water) and 1 mM copper(II) sulphate (50 mM stock in water) were added to the protein samples up to a total reaction volume of 50 μl in the stated order. The samples were incubated for 1 h at room temperature in the dark. The reactions were quenched by acetone precipitation and the protein pellets were resuspended in 2 × gel loading buffer (100 mM Tris–HCl (pH 6.8), 200 mM dithiothreitol, 20% (v/v) glycerol, 4% (w/v) SDS, 0.02% (w/v) bromophenol blue).

### Fluorescence gel imaging

The labelled samples were separated on 15% SDS‐PAGE gels and fluorescently labelled proteins were detected by in‐gel scanning using a Typhoon FLA 9000 scanner at ex488/em520 or ex633/em670 (GE Healthcare UK Ltd, Buckinghamshire, UK). Subsequent to fluorescence imaging, the gels were stained with Coomassie Brilliant Blue R‐250 (Sigma‐Aldrich).

### Large‐scale labelling and pull‐down

*Arabidopsis* leaf extracts or *N. benthamiana* AFs were mixed with 10 μM DK‐01 or DMSO in 50 ml falcon tubes at a 5 ml total reaction volume. The mixtures were loaded in an ice‐cooled 24‐well microplate in four equal parts of 1.25 ml fractions and irradiated at 245 nm using a UV lamp for 30 min on ice. The labelling reactions were quenched by chloroform/methanol precipitation (Wessel & Flügge, 1984). The labelled proteins were processed as described previously except for the replacement of biotin‐azide with biotin‐picolyl‐azide (Morimoto *et al*., 2019).

### Proteomics

Procedures for in‐gel/on‐bead digestion, MS, and the identification of peptides and protein using Max Quant are explained in Methods S1.

### Agromonas assay

Agromonas assays were performed as described previously with minor modifications (Buscaill *et al*., 2021). Agroinfiltrated leaves of *N. benthamiana* at 3 dpi were infiltrated with *Pto*DC3000 at an OD_600_ of 0.001 and plants were kept for 3 d in a growth cabinet at 21°C. Three leaf discs (1 cm in diameter) were excised from infected leaves and each leaf disc (3.2 mm in diameter) was soaked in 15% (v/v) H_2_O_2_ for 1 min to sterilize leaf surfaces. Leaf discs were washed twice in sterile water and ground in sterile water for 5 min using the tissue lyser and metal beads (Biospec Products, Bartelsville, OK, USA). Serial dilutions of the homogenate were plated onto LB agar plates containing Pseudomonas CFC Agar Supplement (5 μg ml^−1^ cetrimide, 5 μg ml^−1^ fucidin, 25 μg ml^−1^ cephalosporin; SR0103; Oxoid Ltd, Basingstoke, UK). Colonies were counted after 36 h of incubation at 28°C. *P*‐values were calculated using the two‐tailed Student *t*‐test to compare bacterial growth between leaves expressing empty vector, PRp27 WT and PRp27 H122F.

## Results

### Hydroxamate‐based probes label distinct proteins in Arabidopsis leaf extracts

To test the utility of the hydroxamate‐based probes in plants, we profiled eight such probes for labelling in Arabidopsis leaf extracts (Fig. 1a; Sieber *et al*., 2006). All the tested probes share four features: a hydroxamate group that binds the zinc atom in metalloproteases; a dipeptide composed of a leucine at position P_1_ and a variable amino acid residue at position P_2_; a benzophenone photo‐cross‐linker for covalent labelling; and an alkyne minitag for coupling with a biotin or a fluorophore using click‐chemistry (Fig. 1a).

To establish labelling in plants, we incubated Arabidopsis leaf extracts with the eight different probes on ice and irradiated the samples with UV for photoaffinity crosslinking. After labelling, we coupled the alkyne‐labelled proteins to an azide‐fluorophore using click‐chemistry (Kaschani *et al*., 2009b). Labelled proteomes were separated on protein gels and detected by in‐gel fluorescence scanning. We detected five signals of 70–130 kDa with most of the eight probes, but that were absent from the no‐probe control (Fig. 1b). Interestingly, three of the probes caused distinct labelling profiles, either by lacking signal 1 (ProP_2_ and GluP_2_) or signals 2 and 3 (PheP_2_) (Fig. 1b). Furthermore, the GlyP_2_ or AsnP_2_ probes caused strong intensities for signals 2–4, whereas LysP_2_ or PheP_2_ probes caused a strong signal 5 (Fig. 1b). Taken together, the differential labelling indicates that the probes have different specificities and that these signals are caused by different proteins. Based on the robust labelling of all five signals, we selected the probe with the P_2_ Gly residue for further experiments.

**Fig. 2:**
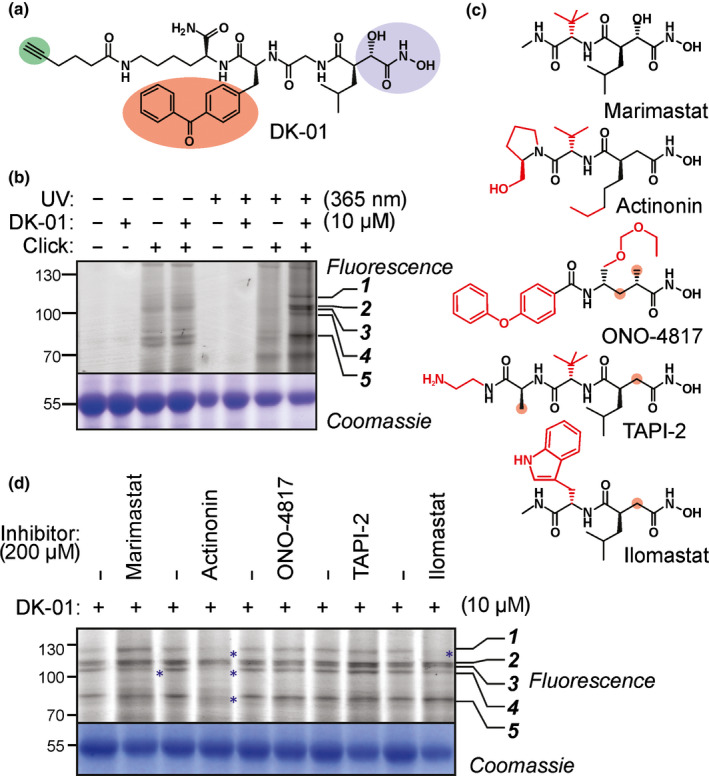
Hydroxamate‐based inhibitors cause differential labelling. (a) Structure of DK‐01, containing a hydroxamate (blue), photoreactive benzophenone (red), alkyne minitag (green) and a dipeptide linker with P_1_ = Leu and P_2_ = Gly. (b) DK‐01 labels several proteins in Arabidopsis leaf extracts and requires both UV treatment and click‐chemistry. (c) Structures of the hydroxamates used, highlighting the differences with DK‐01 in red. (d) Differential labelling upon preincubation with hydroxamates. Leaf extracts of wild‐type Arabidopsis were preincubated with and without 200 µM inhibitors then end‐labelled with or without 10 µM DK‐01 by UV irradiation at 254 nm for 30 min on ice. Click‐chemistry with and without Cy3‐picolyl‐azide was used to couple the alkyne minitag to a fluorophore. Proteins were separated on SDS‐PAGE gels and fluorescent proteins were detected by in‐gel fluorescence scanning.

We resynthesized the GlyP_2_ probe, which was named DK‐01 (Fig. 2a). Labelling Arabidopsis leaf extracts with DK‐01, followed by coupling to a fluorophore via ‘click‐chemistry’, causes the same five signals as we detected with GlyP_2_ (Fig. 2b). Labelling with DK‐01 requires UV treatment at 365 nm (Fig. 2a), consistent with it being a photoaffinity probe. The DK‐01 lane is also darker than the adjacent no‐probe control, which is probably caused by aspecific photocrosslinking with excess probes. However, click‐chemistry in the absence of DK‐01 causes substantial ‘background’ (Fig. 2a), which must originate from aspecific reactivities of Cy3‐picolyl‐azide reagent. We next assessed the characteristics of DK‐01 labelling by testing various labelling times (5–60 min), probe concentrations (0.1–100 μM) and pH (pH 3–9). These experiments showed that DK‐01 labelling occurs within 10 min and reaches the maximum within 30 min, and longer incubations cause background labelling (Fig. S1a). The optimal probe concentration is 10 μM because fewer signals (only signals 2–4) are labelled at lower probe concentrations and background labelling occurs at higher probe concentrations, caused by nonselective photoreactivity labelling by excess probe (Fig. S1b). Labelling also depends on pH (Fig. S1c), consistent with being caused by labelling different proteins. Signals 2, 3 and 5 appeared above pH 5 and intensify at a higher pH, while signals 1 and 4 appeared independently of pH between pH 5 and 8. Collectively, we conclude that DK‐01 labelling for signals 1–5 is optimal at cytonuclear pH (pH 7.4) with 10 µM DK‐01 for 30 min. These conditions were used in further experiments.

Differential labelling with the different probes and at the different conditions indicates that signals 1–5 are caused by labelling different proteins. To explore the properties of these proteins further, we took advantage of commercially available hydroxamates that are used in anticancer therapy because they inhibit MMPs. We selected five different peptidyl hydroxamates (Fig. 2c), and preincubated leaf extracts to determine if they can prevent labelling with DK‐01. Interestingly, whilst ONA‐4817 and TAPI‐2 do not affect labelling, preincubation with marimastat and ilomastat prevents labelling of signals 4 and 1, respectively, whereas preincubation with actinonin prevents labelling of signals 1, 4 and 5 (Fig. 2d). Thus, the differences in moieties in peptidyl hydroxamate inhibitors dictates the selectivity for different proteins. Together, these experiments indicate that DK‐01 labels distinct proteins in Arabidopsis leaf extracts.

### DK‐01 labels a broad range of Arabidopsis metalloproteases

**Fig. 3:**
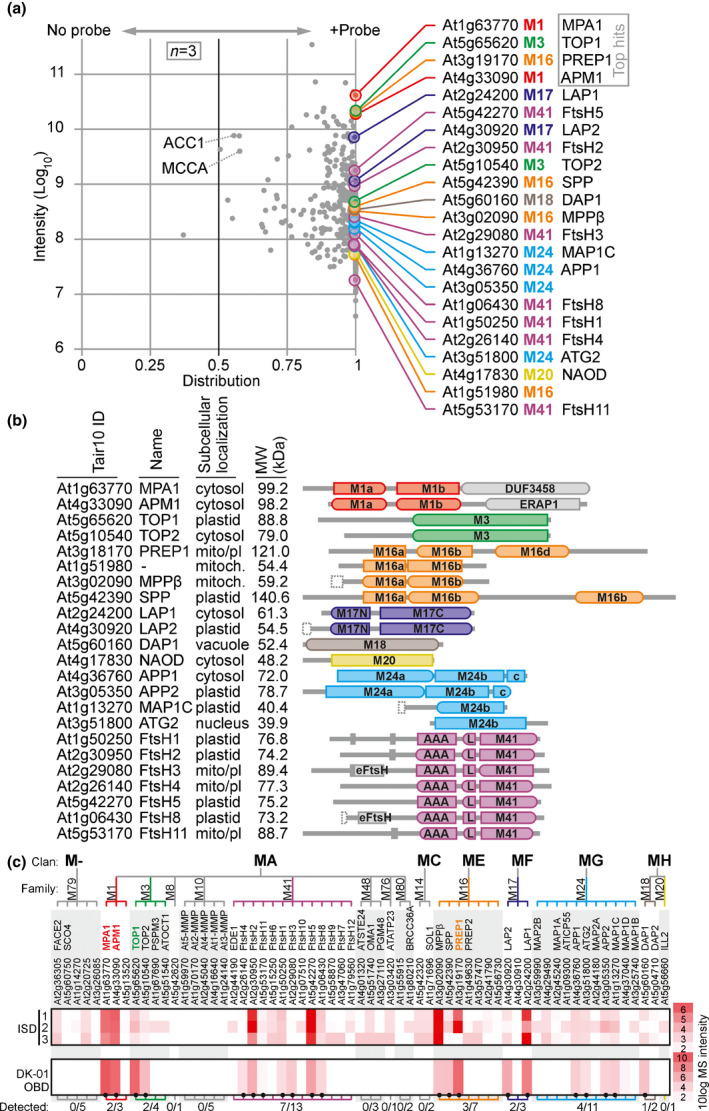
DK‐01 targets a broad range of Arabidopsis metalloproteases. (a) Arabidopsis leaf extracts were labelled with or without 10 µM DK‐01 by UV irradiation at 254 nm for 30 min on ice. Alkyne‐labelled proteins were biotinylated using click‐chemistry, biotinylated proteins were enriched on streptavidin beads and on‐bead digested with trypsin, and released peptides were analysed by LC‐MS/MS. The sum of the MS signal intensities was plotted against the distribution of the MS signal intensities over the no‐probe‐control and the DK‐01‐labelled sample. Only proteins that were detected in all three DK‐01 samples are shown. Twenty‐six enriched proteins are metalloproteases, identified on the right by accession number, subfamily and name. Nonspecific abundant proteins include endogenously biotinylated proteins MCCA and ACC1. (b) Domain structures and classification of the 23 identified metalloproteases. The protein IDs and names are given along with the predicted subcellular localization based on the Arabidopsis Subcellular Database (SUBA), the predicted molecular weight (MW; kDa) and the protein domain structures (PFAMs) summarized as follows: M1a (PF17900); M1b (PF01433); DUF3458 (PF11940/PF17432); ERAP1‐like domain (PF11838); M3 peptidase (PF01432); M16a (PF00675); M16b (PF05193); M16c (PF16187); M16d (PF08367); M17N (PF02789); M17C catalytic domain (PF00883); M18 peptidase (PF02127); M20 peptidase (PF01546); M24a (PF01321/16189); M24b catalytic M24 peptidase (PF00557); c, C‐terminal M24 domain (PF16188); eFtsH, extracellular FtsH domain (PF06484); AAA, ATPase (PF00004); L, ATPase lid (PF17862); and M41 peptidase (PF01434). (c) Most abundant metalloproteases detected in leaf extracts detected by DK‐01 labelling. The protein intensities of all metalloproteases were extracted from the pep2pro database from three independent MS experiments on leaf extracts and compared to the intensities of the metalloproteases detected in the pull‐down upon DK‐01 labelling. The classification of metalloproteases into families and clans is from the MEROPS database.

To identify DK‐01 targets, we performed gel‐free LC‐MS/MS analysis. To purify labelled proteins, DK‐01‐labelled proteins and the no‐probe‐control were coupled to azide‐picolyl‐biotin (Uttamapinant *et al*., 2012) using ‘click‐chemistry’. Biotinylated proteins were enriched on streptavidin beads in triplicate, then on‐bead digested with trypsin/LysC and subjected to LC‐MS/MS analysis. The total intensities for the 816 Arabidopsis proteins detected in all (*n* = 3) DK‐01 replicates were plotted against their average distribution between the no‐probe‐control and DK‐01‐labelled samples (Fig. 3a; Dataset S1). Twenty‐three metalloproteases were consistently and nearly exclusively identified in DK‐01‐labelled samples (> 99.5% enrichment), while some abundant endogenously biotinylated proteins such as ACC1 and MCCA were identified in both the no‐probe‐control and DK‐01‐labelled samples (Fig. 3a). The other proteins were not annotated as metalloproteases. Although some of these proteins might be true DK‐01 targets, many may be the result of nonspecific photoreactivity labelling caused by use of an excess of the probe, consistent with the background seen with fluorescent labelling (Figs 2b, S1b). Other detected proteins may be the result of nonselective click‐chemistry labelling, consistent with the background signals detected in the no‐probe control (Fig. 2b), whilst other detected proteins might stick to beads coated with DK‐01‐labelled proteins, but not to the beads in the no‐probe control. In contrast to most of these other detected proteins, metalloproteases are nearly exclusively found in the DK‐01 samples.

Interestingly, these 23 metalloproteases belong to eight unrelated subfamilies and have remarkably diverse roles (Fig. 3b). These proteases are predicted to localize in different subcellular compartments and have molecular weights ranging from 38.5 to 140 kDa (Fig. 3b). We identified two M1 subfamily proteases: meiotic prophase aminopeptidase‐1 (MPA1) and aminopeptidase M1 (APM1), which act in meiosis and mitosis, respectively (Peer, 2011). We also detected thimet oligopeptidases TOP1 and TOP2 of subfamily M3, which are regulated by the plant stress hormone salicylic acid (SA) and act in defence signalling (Moreau *et al*., 2013). We detected four metalloproteases of subfamily M14, including presequence protease‐1 (PREP1) and stromal processing protease (SPP), which reside in plastids and remove transit peptides upon protein import (Trösch & Jarvis, 2011; Kmiec & Glaser, 2012). We also detected M17 subfamily leucine aminopeptidases LAP1 and LAP2, which remove N‐terminal leucines and other residues from proteins, thereby regulating their abundance via the N‐end rule (Matsui *et al*., 2006). In addition, we detected aspartyl aminopeptidase‐1 (DAP1, subfamily M18), which removes acidic residues from the N‐termini of small peptides (Park *et al*., 2017) and *N*‐acetyl ornithine deacetylase (NAOD, subfamily M20), which breaks the amide bond in *N*‐acetylornithine to release ornithine for amino acid biosynthesis (Molesini *et al*., 2017). We also detected four metalloproteases of subfamily M24, including Met aminopeptidase 1C (MAP1C), which removes the N‐terminal Met residues from nascent proteins (Ross *et al*., 2005). Finally, we detected seven of the 12 known active members of subfamily M41: FtsH1, ‐2, ‐3, ‐4, ‐5, ‐8 and ‐11. These ATP‐dependent zinc metalloproteases are embedded as heterohexamers in the thylakoid membrane with the catalytic domain in the stroma, where they control protein homeostasis, remove damaged photocentres and regulate plastid development (Kato & Sakamoto, 2018). In summary, DK01‐enriched metalloproteases are biologically and biochemically diverse enzymes, located at different subcellular compartments.

To investigate if DK‐01 labelling correlates with protein abundance of metalloproteases in leaf proteomes, we extracted data for leaf protein intensities from three independent proteomic datasets at the pep2pro database (Baerenfaller *et al*., 2011; ISD, in‐solution digests), and aligned these this to the average protein intensity detected in our pull owns (OBD, on‐bead digest). Importantly, there was a strong correlation between protein accumulation in leaves, and the detection of these proteins upon DK‐01 labelling (Fig. 3c), indicating that DK‐01 is not selective for specific metalloprotease families, but can label the majority of the Arabidopsis metalloproteases. This implies that these other metalloproteases will be detected upon DK‐01 labelling when present sufficiently in a proteome.

### Four metalloproteases are responsible for the five high molecular weight (MW) signals

The protein intensities from the on‐bead digest indicate that the main targets of DK‐01 in Arabidopsis leaf extracts are PREP1, MPA1, APM1 and TOP1, which have predicted MWs of 121.0, 99.2, 98.2 and 88.9 kDa, respectively. To test if these metalloproteases cause the signals at 90–120 kDa, we separated the purified biotinylated proteins on a protein gel and excised the bands to perform an in‐gel digest. Metalloprotease PREP1 (At3g18170) was predominantly identified from band 1, and metalloproteases MPA1 (At1g63770) and APM1 (At4g33090) were top hits for bands 2 and 3 (Fig. S2; Dataset S1). Bands 4 and 5 were not analysed.

**Fig. 4:**
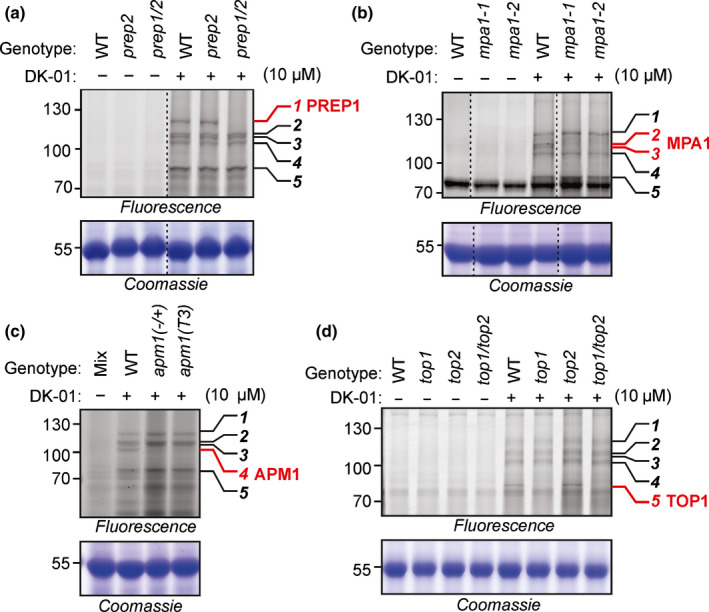
Knockout mutants deconvolute high‐MW metalloprotease signals. (a) Metalloprotease PREP1 is required for signal 1. (b) MPA1 is required for signals 2 and 3. (c) APM1 is required for signal 4. (d) TOP1 is required for signal 5. Leaf extracts of wild‐type (WT) and mutant plants were labelled with or without 10 µM DK‐01 by UV irradiation at 254 nm for 30 min on ice. Proteins were separated on SDS‐PAGE gels and fluorescent proteins were detected by in‐gel fluorescence scanning at ex488/em520 or ex633/em670. Five specific signals are numbered on the right. Coomassie stains are provided as loading controls.

To further define the identities of the metalloproteases causing signals 1–5, we collected mutants lacking the four top candidates PREP1, MPA1, APM1 and TOP1 from various sources (see the Materials and Methods section) and tested leaf extracts for altered DK‐01 labelling. Signal 1 was absent in the *prep1/prep2* double mutant, but not in the *prep2* single mutant, whilst the *prep1* mutant did not germinate (Fig. 4a). We conclude that signal 1 is caused by PREP1, which is consistent with the detection of only PREP1 in our in‐gel digest experiment. Signals 2 and 3 are absent from the *mpa1‐1* and *mpa1‐2* single mutants (Fig. 4b), indicating that MPA1 occurs as two labelled isoforms causing signals 2 and 3. Signal 4 is reduced in the heterozygous *apm1* mutant (+/−) and in the *apm1* mutant line transformed with human insulin‐regulated amino‐peptidase (IRAP; Hosein *et al*., 2010; Fig. 4c, lane T3), indicating that signal 4 is caused by APM1. Homozygous *apm1*(−/−) mutants cannot be tested because these seedlings die at day 5 (Peer *et al*., 2009). Finally, signal 5 is absent in the *top1* single mutant as well as the *top1/top2* double mutant, but not the *top2* single mutant (Fig. 4d), indicating that TOP1 causes signal 5, consistent with TOP1 being much more abundant in the MS experiment (Fig. 3a).

The detected MW of signals 1–5 corresponds to the predicted MW of the labelled proteins: PREP1 is predicted at 121 kDa and was found at 120 kDa (signal 1); MPA1 is predicted at 99 kDa and was found at 105 kDa (signals 2 and 3); APM1 is predicted at 98 kDa and was found at 100 kDa (signal 4); and TOP1 is predicted at 88 kDa and was found at 80 kDa (signal 5). In conclusion, analysis of the mutants revealed the identities of signals 1–5, and these correspond with predicted MWs, with the top candidates detected by in‐solution digests and with the proteins detected with in‐gel digests.

### PRp27 is differentially labelled in apoplast upon infection

Having established metalloprotease profiling, we applied this in a biological research context. To investigate the potential differential activity of metalloproteases in the apoplast of *N. benthamiana* during pathogen challenge, we tested DK‐01 labelling on apoplastic fluids isolated from leaves of *N. benthamiana*, 2 d after infiltration with water (Mock control), wild‐type *P. syringae* pathovar *tomato* DC3000 (*Pto*DC3000 WT) and derived mutants lacking the type‐3 secretion system (T3SS, Δ*hrpA*), or lacking the T3 effector hopQ1‐1 (*ΔhQ*). In *N*. *benthamiana*, the WT bacteria activate effector‐triggered immunity (ETI) because the hopQ1‐1 effector is recognized by the *Roq1* resistance gene product (Schultink *et al*., 2017). By contrast, the Δ*hrpA* mutant activates pattern‐triggered immunity (PTI) and the *ΔhQ* mutant is not recognized and causes disease because it lacks hopQ1‐1.

**Fig. 5:**
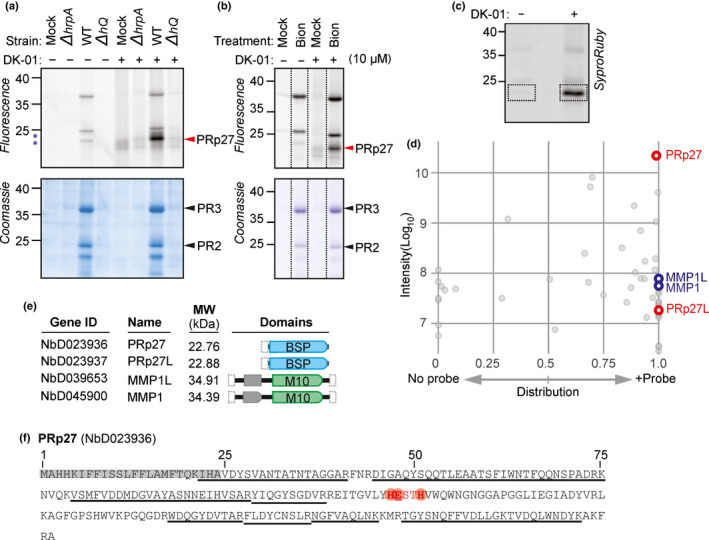
Differential labelling of PRp27 upon infection with WT *Pto*DC3000. (a) Treatment with WT *Pto*DC3000 induces a DK‐01‐labelled protein. *Nicotiana benthamiana* leaves were infiltrated with water (Mock), WT *Pto*DC3000, and the derived *ΔhrpA* and *ΔhQ* mutants. Apoplastic fluids (AFs) were isolated at 2 d postinfiltration (2 dpi) and labelled with or without 10 µM DK‐01 by UV irradiation at 254 nm for 30 min on ice. Alkyne‐labelled proteins were coupled to Cy3‐picolyl‐azide using click‐chemistry. Proteins were separated on SDS‐PAGE gels and fluorescent proteins were detected by in‐gel fluorescence scanning at ex488/em520. The specific signal at 23 kDa and the PR proteins are indicated with red and black arrowheads, respectively. (b) The 23 kDa signal is induced by SA signalling. *Nicotiana benthamiana* leaves were infiltrated with water or 2 mM Bion. Apoplastic fluids were isolated at 4 dpi and labelled with or without 10 µM DK‐01 by UV irradiation at 254 nm for 30 min on ice. Alkyne‐labelled proteins were coupled to Cy5‐picolyl‐azide using click‐chemistry. Proteins were separated on SDS‐PAGE gels and fluorescent proteins were detected by in‐gel fluorescence scanning at ex633/em670. (c) Analysis of the purified 23 kDa gel region. Apoplastic fluids from WT infiltrated plants were labelled with and without DK‐01 and coupled to biotin. Biotinylated proteins were purified, separated on gels and stained with Sypro Ruby (top). The 20–25 kDa region was excised and incubated with trypsin/LysC, and released peptides were analysed by MS. (d) Distribution graph of proteins identified from 20 to 25 kDa gel slices. The sum of the peptide intensities was plotted against the distribution over the probe and no‐probe control for each protein. Four highly enriched proteins include pathogenesis‐related protein 27 (PRp27), PRp27‐like protein (PRp27L), matrix metalloprotease 1 (MMP1) and MMP‐1‐like protein (MMP1L), highlighted in red or blue, respectively. (e) Domain structures of the four proteins highlighted in (c). The protein IDs, names, predicted MWs (kDa) not including N‐ and C‐terminal signal peptides, and the PFAM protein domains: BSP (PF04450), M10 (PF00413) and PG binding 1 (PF01471) are given. (f) PRp27 contains the consensus zinc‐binding motif HExxH. The predicted signal peptides (grey) and the HExxH motif (red) are highlighted. The identified peptides (underlined) cover *c*. 61% of the PRp27 protein sequence.

Interestingly, labelling of apoplastic fluids with DK‐01 revealed distinct labelling profiles depending on the treatment. A unique, strong 23 kDa signal appears upon infection with WT bacteria, but not with the Δ*hrpA* or *ΔhQ* bacteria or the Mock control (Fig. 5a). Weaker signals are also displayed at 25 and 37 kDa only upon WT treatment, but these signals are also displayed in the absence of DK‐01 (Fig. 5a) and seem to originate from nonspecific labelling of highly abundant pathogenesis‐related (PR) proteins during click‐chemistry. Coomassie staining indeed displayed the accumulation of PR proteins PR2 and PR3 in the plant apoplast upon WT infection, consistent with a strong ETI response. This indicates that accumulation of the 23 kDa labelled protein associates with PR proteins.

To further test the association with PR protein accumulation, we treated plants with Bion (Syngenta, Milano, Italy), which contains an analogue of SA, which is known to induce PR protein accumulation in tobacco (Lavergne *et al*., 2017). DK‐01 labelling on apoplastic fluids isolated from Bion‐treated plants showed the 23 kDa labelled protein, concurrently with PR2 and PR3 accumulation (Fig. 5b), indicating that the signal at 23 kDa is caused by a distinct PR protein.

To identify the protein causing the 23 kDa signal, DK‐01‐labelled proteins were coupled to biotin by click reaction, purified, separated by gel electrophoresis and stained with Sypro Ruby; the 20–25 region of the gel was excised and subjected to in‐gel trypsin/LysC treatment, followed by MS analysis (Fig. 5c). The intensity of the identified proteins was plotted against their distribution between the no‐probe control and the DK‐01‐labelled samples. The strongest ion intensity corresponds to PRp27 (NbD023936), which was predominantly identified in the DK‐01‐labelled sample (Fig. 5d; Dataset S2), with peptides covering *c*. 61% of the protein sequence (Fig. 5f). PRp27 is a PR protein (PR‐17; Xie & Goodwin, 2009) and its predicted MW without signal peptide is 22.76 kDa, which is consistent with the 23 kDa of the observed labelled protein. PRp27 is not currently classified as a metalloprotease in the MEROPS database of proteolytic enzymes (Rawlings *et al*., 2018), but it contains a zinc‐binding motif (HESTH; Fig. 5e), and PFAM (El‐Gebali *et al*., 2019) assigns this protein as a basic secretory protein (BSP, PF04450).

In addition to PRp27, we also found a PRp27‐like protein (PRp27L = NbD023937) with much weaker ion intensity, and two MMPs (MMP1 = NbD045900 and MMP1L = NbD039653) of the M10 subfamily (Fig. 5d,e). Although these two MMPs are predicted to have an MW of 35 kDa upon removing N‐ and C‐terminal signal peptides, plant MMPs are often processed further until the 25 kDa catalytic domain (Marino *et al*., 2014).

### Putative active site of PRp27 is required for labelling but not for immunity

**Fig. 6:**
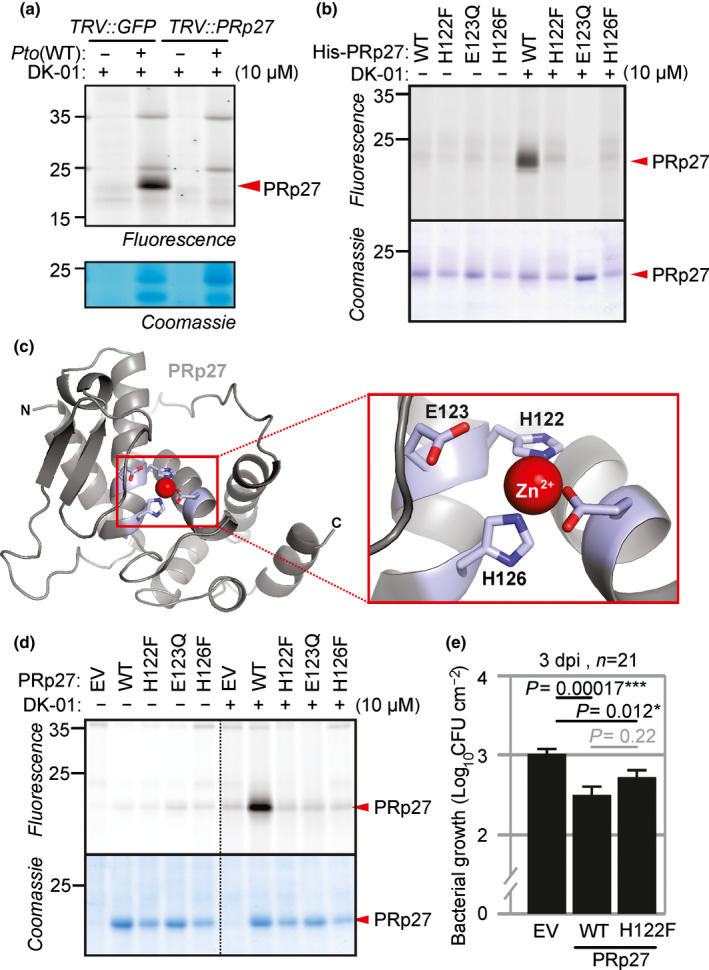
Putative active site of PRp27 is required for labelling but not plant immunity. (a) PRp27 depletion by VIGS removes the 23 kDa signal. Apoplastic fluids (AFs) isolated from the *TRV::GFP* and *TRV::PRp27* leaves infected with *Pto*DC3000 (WT) or water were labelled with or without 10 µM DK‐01 by UV irradiation at 254 nm for 30 min on ice. Alkyne‐labelled proteins were fluorescently coupled to Cy3‐picolyl‐azide using click chemistry. Proteins were separated on SDS‐PAGE gels and fluorescent proteins were detected by in‐gel fluorescence scanning at ex488/em520. (b) Amino acid substitution at the putative zinc‐binding sites abolishes DK‐01 labelling of PRp27 expressed in *E. coli*. Extracts of *E. coli* expressing WT and H122F, E123Q and E126F mutant His‐PRp27 proteins were labelled with 10 µM DK‐01 as described in (a). (c) A three‐dimensional model of the PRp27 structure shows the putative architecture of the zinc‐binding domain in PRp27, coordinated by four amino acid residues. The model was generated with swiss model using PDB 1z5h as a template. (d) DK‐01 labels transiently expressed PRp27 (WT), but not the H122F, E123Q or H126F mutants. Apoplastic fluids isolated at 4 dpi from agroinfiltrated leaves coexpressing the empty vector (EV) or PRp27 (WT, H122F, E123Q and E126F) with silencing inhibitor P19 were labelled, with or without 10 µM DK‐01, as described in (a). (e) Both PRp27 WT and the H122F mutant increase immunity to *Pto*DC3000(WT). Agroinfiltrated leaves expressing the empty vector (EV), PRp27(WT) or PRp27(H122F) were infiltrated with *Pto*DC3000(WT) at 2 dpi and bacterial growth was measured 3 d later. Error bars represent mean ± SE of *n* = 21 biological replicates. The experiment shown is representative of three experimental replicates. The *P*‐value was calculated using the two‐tailed Student *t*‐test to compare bacterial growth between leaves expressing empty vector, PRp27 WT and PRp27 H122F. *, *P* < 0.05; ***, *P* < 0.001.

To confirm that the 23 kDa signal is caused by PRp27, we depleted PRp27 by VIGS using Tobacco Rattle Virus (TRV) vectors (Lu *et al*., 2003). Importantly, the 23 kDa signal is absent when labelling apoplastic fluids of *TRV:PRp27* but not *TRV:GFP* plants treated with WT *Pto*DC3000 (Fig. 6a). The absence of labelling upon PRp27 depletion, together with the in‐gel digest dataset and the predicted MW and inducibility as a PR protein, supports the conclusion that the 23 kDa signal is caused by PRp27.

To validate whether PRp27 can be labelled by DK‐01, we produced recombinant N‐terminally His‐tagged PRp27 without its native signal peptide in *E. coli*. Labelling extracts of His‐PRp27‐expressing *E. coli* cultures with DK‐01 causes appearance of a strong 23 kDa band corresponding to PRp27 (Fig. 6b), confirming that PRp27 can be labelled with DK‐01.

We next included three substitutions in the HExxH motif that should disrupt zinc binding: H122F, E123Q and H126F. The importance of these residues in coordinating the zinc ion is illustrated with a model of the PRp27 structure, which was generated based on the crystal structure of Tricorn interacting factor F3 (1z5h; Kyrieleis *et al*., 2005; Fig. 6c). This metalloprotease binds a zinc ion in its active site via the HExxH motif (Kyrieleis *et al*., 2005). Labelling extracts of His‐PRp27‐expressing *E. coli* cultures with DK‐01 causes a strong 23 kDa for the WT enzyme, but not for the mutants (Fig. 6b), indicating that PRp27 labelling is dependent on its ability to bind zinc.

We also tested the DK‐01 labelling of PRp27 and its mutants (H122F, E123E and H126F) upon transient overexpression in *N*. *benthamiana* by agroinfiltration. Consistent with the result with the recombinant proteins expressed in *E.coli*, DK‐01 labelled WT PRp27 but not the mutants (Fig. 6d). Coomassie staining indicated that all mutants accumulate as stable proteins in the apoplast. Together, these results show that DK‐01 labelling of PRp27 requires an intact zinc binding motif.

To test the role of putative catalytic activity of PRp27 in defence, we transiently overexpressed PRp27 and its H122F mutant and infected this tissue with *Pto*DC3000, using the recently introduced ‘agromonas’ method (Buscaill *et al*., 2021). Importantly, growth of *Pto*DC3000 in PRp27‐expressing tissue is reduced (Fig. 6e), demonstrating that PRp27 accumulation can retard *Pto*DC3000 growth during defence. Unexpectedly, however, *Pto*DC3000 growth is also significantly reduced by the H122F mutant (Fig. 6e), suggesting that the accumulation of PRp27 protein itself contributes to immunity irrespective of its zinc‐dependent activity.

## Discussion

We have established broad‐range metalloprotease profiling in plants by photoaffinity labelling with the hydroxamate‐based probe DK‐01. We identified 23 metalloproteases representing nine different families with on‐bead digests of DK‐01‐labelled proteins from Arabidopsis leaf extracts, in addition to another 535 enriched proteins. We also used Arabidopsis protease knock‐outs and in‐gel digests to confirm the identity of five high‐MW metalloproteases causing fluorescence signals on protein gels. We subsequently discovered induced labelling of a secreted protein upon bacterial infection of *N. benthamiana* and identified this protein as PRp27, a metalloprotein containing an HExxH motif. We showed that the HExxH motif is required for DK‐01 labelling and that PRp27 suppresses *P. syringae* growth *in planta*, but independently of an intact HExxH motif.

### Broad‐range metalloprotease profiling

By using the DK‐01 probe to detect 23 metalloproteases representing nine different families in Arabidopsis leaf extracts, we have established broad‐range metalloprotease profiling in plants. When these hydroxamate‐based probes were originally tested, 12 proteases representing seven families were identified by LC‐MS from three different mammalian proteomes (Sieber *et al*., 2006). Labelling of another 11 metalloproteases was confirmed using recombinant proteins, including representatives of two additional families. We now detected 23 proteases from a single proteome and expanded the detected families by detecting representatives from the M3, M20 and M24 protease families. There are several explanations for this expansion. First, MS techniques have improved since 2006, facilitating an efficient and sensitive detection of purified proteins. Second, we used picolyl‐azide to perform click‐chemistry reactions more efficiently than in previous work (Uttamapinant *et al*., 2012). Furthermore, higher plant leaf extracts may contain a larger diversity and concentration of metalloproteases when compared to previously used mammalian extracts.

The fact that we detected ‘only’ 23 of the predicted 131 metalloproteases encoded by Arabidopsis genome has several possible explanations. First, many of these proteases may not accumulate to sufficiently high levels to be detected in total leaf extracts. This can, for instance, be because the encoding genes are expressed under specific conditions or the proteins are concentrated at specific subcellular locations. Indeed, PRp27 labelling was drastically induced upon pathogen infection, and MMPs were not detected in leaf extracts, but were detected in apoplastic fluids. More metalloproteases will probably be detected when profiling other organs (e.g. flowers, seeds, roots) and subproteomes (e.g. membranes, nuclei). Second, some metalloproteases may not be targeted by the probe. There will certainly be metalloproteases that cannot bind DK‐01, for instance because they cannot accommodate the Leu residue that resides at the P1 position. These proteins might still be labelled with other hydroxamate derivatives. Finally, some metalloproteases may not be active under the tested conditions. Buffer choice during cell homogenization equalizes cellular conditions such as pH, redox and ion concentrations and this will certainly result in suboptimal conditions for labelling of some metalloproteases. However, assuming that, under optimal conditions, the method can label all proteases within each subfamily for which we have detected representatives (i.e. 79 metalloproteases: 4 × M1, 5 × M3, 7 × M10, 14 × M16, 4 × M17, 2 × M18, 14 × M20, 13 × M24 and 16 × M41), we have already demonstrated the potential of detecting 60% (79/131) of the Arabidopsis metalloproteases.

Because it seems likely that DK‐01 can be used to label most of the plant proteases, this probe can be used to study metalloproteases in different organelles (e.g. nuclei, chloroplasts), in different organs (e.g. roots, seeds) and in different plant species, similar to our studies of Arabidopsis leaf extracts and *N. benthamiana* apoplastic fluids. Specific metalloproteases can be studied as purified proteins or upon transient overexpression, similar to how we studied PRp27. Metalloprotease labelling therefore has a great potential to study metalloproteases in plants.

### High‐MW metalloproteases have different labelling properties

This project sparked off with the observation that high‐MW signals appeared upon labelling Arabidopsis leaf extracts with hydroxamate probes carrying varied residues at the P2 position. Through MS analysis of on‐bead and in‐gel digests, it became clear that these high‐MW signals are caused by labelling of PREP1, MPA1, APM1 and TOP1 by the probes, because of the relatively high ion intensities obtained for these proteins and the matching predicted MWs. Indeed, using Arabidopsis knockout lines, we detected the depletion of: signal 1 in the absence of PREP1, signals 2 and 3 in the absence of MPA1, signal 4 in the absence of APM1, and signal 5 in the absence of TOP1, consistent with their predicted MWs. Interpretation of signals 1–5 detected in Figs 1, 2d and S1 therefore indicates that PREP1 is actinonin/ilomastat‐sensitive and does not accommodate Pro or Glu residues at the P2 position, that MPA1 cannot accommodate Phe at the P2 position and is only active at neutral pH, and that APM1 is marimastat/actinonin‐sensitive and active at pH5 and above, whilst TOP1 labelling is actinonin‐sensitive and requires a neutral pH. Thus, by knowing the origin of these signals, it is now possible to characterize the sensitivity of these proteases to inhibitors, and characterize their labelling at various conditions, in different organs and in various subcellular proteomes. Thus, this work provides robust procedures for the further characterization of these and other metalloproteases.

### Immune‐responsive metalloprotein PRp27 acts in immunity

We discovered a strongly increased labelling of PRp27 by DK‐01 in the apoplast of *N. benthamiana* upon infection with *Pto*DC3000 (WT). We confirmed PRp27 labelling by DK‐01 by the fact that the signal was depleted in *TRV::PRp27* plants, and that PRp27 was labelled with DK‐01 when expressed in *E. coli* and when overexpressed by agroinfiltration. Labelling with DK‐01 is consistent with the presence of an HExxH motif in PRp27, and we confirmed the relevance of this motif by showing that the H122F, E123Q and H126F mutants of PRp27 can no longer be labelled by DK‐01 even though they accumulate stable proteins upon expression in *E. coli* and by agroinfiltration. This implies that DK‐01 labelling indeed depends on the presence of a metal ion bound to the HExxH motif in PRp27.

The induced labelling of PRp27 upon infection correlates with its induced accumulation in the apoplast as a response to infection by WT *Pto*DC3000, which triggers a strong immune response (Wei *et al*., 2007). We detected a similar induced labelling upon Bion treatment, which induces SA signalling as it contains the SA analogue benzothiadiazole (BTH). These data are consistent with the earlier observations that *PRp27* transcription is induced in *N. benthamiana* upon treatment with Actigard, a plant defence activator from Syngenta, which also contains BTH, and upon infection with *P*. *syringae* pv *tabaci* (Xie & Goodwin, 2009). Homologues of *PRp27* are also induced upon biotic stress in other plant species. In tobacco, transcript levels of *NtPRp27* are induced upon wounding and TMV infection (Okushima *et al*., 2000). In wheat, transcripts of the *PRp27* homologue *WCI‐5* are induced upon BTH treatment and upon infection with powdery mildew (Görlach *et al*., 1996), and in barley, transcripts and proteins of the *PRp27* homologue *Hv‐PR17* are induced by powdery mildew infection (Christensen *et al*., 2002). In conclusion, PRp27 is a PR protein with clear homologues in both monocot and eudicot plants.

We found that overexpression of PRp27 reduces bacterial growth of *Pto*DC3000 (WT). These data are consistent with the observation that depletion of PRp27 from *N. benthamiana* by VIGS resulted in an increased susceptibility to *P*. *syringae* pv *tabaci* (Xie & Goodwin, 2009). However, the mechanism by which PRp27 promotes immunity is not yet clear. The presence of an HExxH motif suggested that *Hv*‐PR17 might be an MA‐clan metalloprotease, but no cleavage by *Hv*‐PR17 of casein or gelatin, which are sensitive to a broad range of proteases, was detected (Christensen *et al*., 2002). These observations suggest that *Hv*‐PR17 might hydrolyse specific peptides or small molecules. PRp27 may act in immunity by directly harming the pathogen, or by releasing elicitors from the pathogen that are detected by the plant, or by being a component of possible extracellular immune signalling pathways. How PRp27 and its homologues act in immunity will be the subject of future studies.

## Author contributions

KM designed and performed most of the experiments and wrote the manuscript with RALvdH; DK synthesized DK‐01; FK performed initial labelling with hydroxamates; DH‐W helped establish the labelling conditions; AG performed VIGS and agromonas assays; PB made *TRV::PRp27*; SM performed MS experiments; SAS provided initial hydroxamate probes; BFC supported initial labelling experiments; CJS supported DK‐01 synthesis; RALvdH supervised the project and wrote the manuscript with KM.

## Supporting information


**Dataset S1** Arabidopsis proteins identified by on‐bead‐digest and in‐gel‐digest.

**Dataset S2** *Nicotiana benthamiana* proteins identified by in‐gel‐digest.

**Fig. S1** Characteristics of DK‐01 labelling in Arabidopsis leaf extract.
**Fig. S2** High‐MW signals contain metalloproteases.

**Methods S1** Experimental methods for labeling in Fig. 1, DK‐01 synthesis and MS analysis.

**Table S1** Plasmids.
**Table S2** Oligonucleotides.
Please note: Wiley Blackwell are not responsible for the content or functionality of any Supporting Information supplied by the authors. Any queries (other than missing material) should be directed to the *New Phytologist* Central Office.

## Data Availability

The MS proteomics data have been deposited with the ProteomeXchange Consortium via the PRIDE partner repository (Perez‐Riverol *et al*., 2022), with dataset identifier PXD032265.
