# Supplementary material for: Broad‐range metalloprotease profiling in plants uncovers immunity provided by defence‐related metalloenzyme
Source: New Phytol. 2022 May 26;235(3):1287–301. doi: 10.1111/nph.18200 (PMC9322406; doi:10.1111/nph.18200)
Supplement: Supplementary file 3 — Fig. S1 Characteristics of DK‐01 labelling in Arabidopsis leaf extract. Fig. S2 High‐MW signals contain metalloproteases. [file NPH-235-1287-s003.pdf]

New Phytologist Supporting Information

Broad-range metalloprotease profiling in plants uncovers immunity provided by defence-related metalloenzyme

Kyoko Morimoto, Daniel Krahn, Farnusch Kaschani, Digby Hopkinson-Woolley, Anna Gee, Pierre Buscaill, Shabaz Mohammed, Stephan A. Sieber, Benjamin F. Cravatt, Christopher J. Schofield, Renier A. L. van der Hoorn

Article acceptance date 14 April 2022

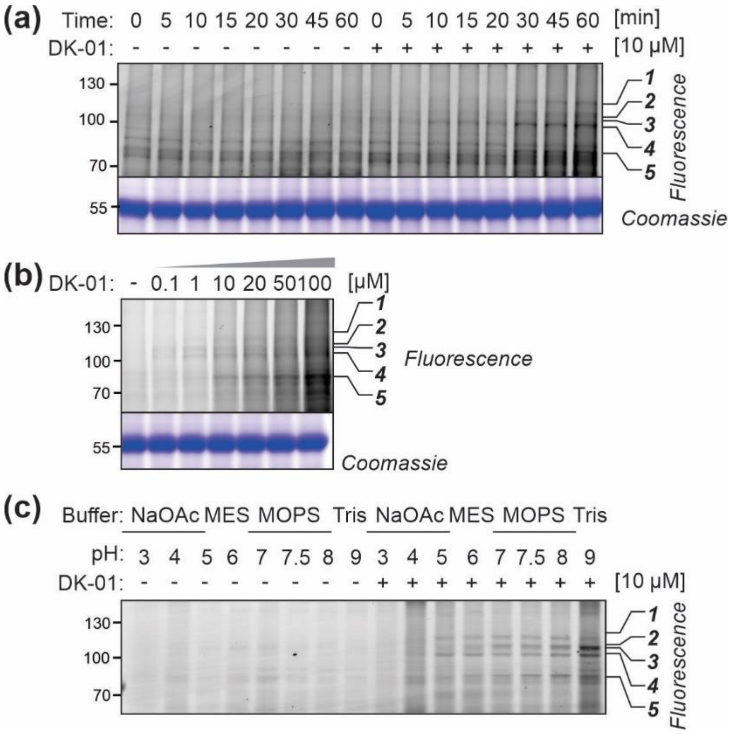

Fig. S1 Characteristics of DK-01 labelling in Arabidopsis leaf extract

(a) Time-course of DK-01 labelling. Arabidopsis leaf extracts were labelled with 10 µM DK-01 by UV irradiation at 254 nm for various time incubation times (0-60 min).

(b) Concentration-dependency of DK-01 labelling. Arabidopsis leaf extracts were labelled with various concentrations of (0.1-100 µM) DK-01.

(c) pH-dependency of DK-01 labelling. Arabidopsis leaf proteomes were extracted in water, buffered to various pH values and labelled with 10 µM DK-01.

(a-c) Arabidopsis leaf extracts were labelled at pH 7.4 with 10 µM DK-01 by UV irradiation at 254 nm for 30 min on ice, unless stated otherwise. Alkyne-labelled proteins were fluorescently coupled to a fluorophore using click chemistry with Cy3-picolyl-azide. Proteins were separated on SDS-PAGE gels and fluorescent proteins were detected by in-gel fluorescence scanning at ex488/em520. Coomassie staining provides the loading control.

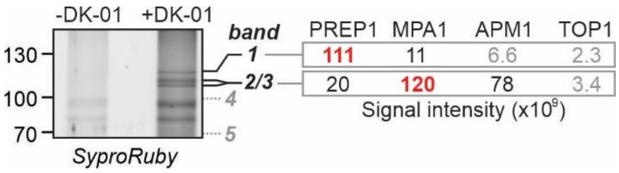

Fig. S2 High MW signals contain metalloproteases.

Arabidopsis leaf extracts were labelled with or without 10 µM DK-01. Alkyne-labelled proteins were biotinylated using click chemistry with biotin-picolyl-azide. Biotinylated proteins were purified and separated on protein gel and stained with Sypro Ruby. Gel slices were excised and treated with trypsin and Lys-C and released peptides were analysed by LC-MS/MS. The top-4 proteins as identified by MS intensities are shown.
