# Supplementary material for: Broad‐range metalloprotease profiling in plants uncovers immunity provided by defence‐related metalloenzyme
Source: New Phytol. 2022 May 26;235(3):1287–301. doi: 10.1111/nph.18200 (PMC9322406; doi:10.1111/nph.18200)
Supplement: Supplementary file 4 — Methods S1 Experimental methods for labeling in Fig. 1, DK‐01 synthesis and MS analysis. [file NPH-235-1287-s004.pdf]

New Phytologist Supporting Information

Broad-range metalloprotease profiling in plants uncovers immunity provided by defence-related metalloenzyme

Kyoko Morimoto, Daniel Krahn, Farnusch Kaschani, Digby Hopkinson-Woolley, Anna Gee, Pierre Buscaill, Shabaz Mohammed, Stephan A. Sieber, Benjamin F. Cravatt, Christopher J. Schofield, Renier A. L. van der Hoorn

Article acceptance date 14 April 2022

Supporting Methods S1

Labelling procedure for Figure 1 - Rosettes of 5-week-old *Arabidopsis* plants grown under the short-day condition were ground using a mortar and pistil on ice. Total proteins were extracted in the extraction buffer (1x PBS) equivalent to a half volume of the fresh weight of plant tissues. The extracts were filtered through a 0.22 µm sterile filter. The protein concentration of the flowthrough was measured using the RC/DC protein assay kit (Biorad) and adjusted to 2 mg/mL with extraction buffer. The 49 µL of resulting extracts were mixed with 1 µL of 50 µM of the hydroxamate probes or DMSO. The mixtures were loaded in an ice-cooled flat-bottom 96-well microplate in 50 µL fractions and irradiated at 366 nm for 30 min using a UV-lamp (Konrad Benda, Laborgeräte und Ultraviolettstrahler, Typ NU-8KL). The labelling reactions were transferred to a 1.5 mL microtube and click-chemistry reactions were performed by adding 20 µM Rhodamine (Rh) -azide, 100 µM TBTA, 2 mM TCEP and 1 mM copper(II) sulfate to the reactions. The samples were incubated for 1 hour at RT in the dark. The reactions were quenched by adding 15 µL of 4 × Laemmli solubilization buffer. The labelled samples were separated on large 10% SDS-PAGE gels and fluorescently labelled proteins were visualized by in-gel fluorescent scanning using a Hitachi FMBio Ile flatbed scanner (MiriaBio).

DK-01 synthesis - DK-01 was synthesised as follows: The synthesis was carried out according to a general solid phase peptide synthesis procedure, following an Fmoc-protection strategy. Couplings were conducted in a syringe reactor using the corresponding Fmoc-amino acids (3 eq.), hydroxybenzotriazole (HOBt) (3 eq.), dicyclohexylcarbodiimide (DIC) (3 eq.) with a reaction time of 45 min (rt, agitation) per coupling step. A Fmoc-Lys(Mtt)-OH loaded rink amide resin (375 mg, 0.19 mmol, loading 0.5 mmol/g) was utilized. After Mtt-side chain deprotection (2% CF_3CO_2H in CH_2Cl_2 , 2 min incubation), 5-hexynoic acid (64 mg, 0.56 mmol, 3 eq.) was coupled (2x, double coupling). The Fmoc-group was removed utilizing a solution of 5% diethylamine in DMF (rt, agitation, 15 min, 2x). Following the same procedure, Fmoc-BPA-OH (275 mg, 0.56 mmol, 3 eq.), Fmoc-Gly-OH (166 mg, 0.56 mmol, 3 eq.) and pre-synthesised (*R*)-2-((*S*)-2,2-dimethyl-5-oxo-1,3-dioxolan-4-yl)-4-methylpentanoic acid (Qui et al., 2009) (128 mg, 0.56 mmol, 3 eq.) were coupled. The crude product was cleaved from the resin (100% CF_3CO_2H, rt, agitation, 1h) and purified by reversed phase HPLC (H_2O:ACN, 0.1%CF_3CO_2H; gradient: 3 → 80%). The desired product was obtained a colourless powder. **Yield**: 13.8 mg (18.8 µmol). **LC-MS** (ESI^+) *m/z*: $[M-H]^+$ 735.1; **HRMS** (ESI^+) *m/z*: $[M+H]^+$ Calcd for $C_{38}H_{51}N_6O_9^+$ 735.37120; found 735.37067.

In-gel / on-bead digestion and MS - For in-gel digestions, gel bands were excised and treated with Trypsin/Lys-C Mix (Promega) as described with minor modifications (Shevchenko et al., 2006). On-bead digestions were performed as described with minor modifications (Weerapana et al., 2007). Peptides were separated on an Ultimate 3000 UHPLC system (Thermo Fisher) and electrosprayed directly into a QExactive mass spectrometer (Thermo Fisher) The peptides were trapped on a C18

PepMap100 pre-column (300 µm i.d. x 5 mm, 100 Å, Thermo Fisher) using solvent A (0.1% formic acid in water) at a pressure of 500 bar, then separated on an in-house packed analytical column (75 µm i.d. x 50 cm packed with ReproSil-Pur 120 C18-AQ, 1.9 µm, 120 Å, Dr. Maisch GmbH). Data were acquired in a data-dependent mode (DDA). Full scan MS spectra were acquired in the Orbitrap (scan range 350-1500 m/z, resolution 70000, AGC target 3e6, maximum injection time 50 ms). The 10 most intense peaks were selected for HCD fragmentation at 30% of normalised collision energy at resolution 17500, AGC target 5×10^4, maximum injection time 120 ms with first fixed mass at 180 m/z. Charge exclusion was selected for unassigned and 1+ ions.

Peptide and Protein Identification using MaxQuant - RAW spectra were submitted to an Andromeda (Cox et al. 2011) search in MaxQuant (1.6.6.0) using the default settings (Cox & Mann 2008). Label-free quantification and match-between-runs was activated (Cox et al. 2014). For Arabidopsis experiments the MS/MS spectra data were searched against the Uniprot *A. thaliana* reference database (uniprot-proteome_UP000006548.fasta, 33,447 entries, downloaded 3/29/2016) for Arabidopsis and the *N. benthamiana* reference database (NbDE proteome (74,802 entries; Kourelis et al., 2019). All searches included a contaminants database search (as implemented in MaxQuant, 245 entries). The contaminants database contains known MS contaminants and was included to estimate the level of contamination. Andromeda searches allowed oxidation of methionine residues (16 Da) and acetylation of the protein N-terminus (42 Da) as dynamic modifications and the static modification of cysteine (57 Da, alkylation with iodoacetamide). Enzyme specificity was set to “Trypsin/P” with two missed cleavages allowed. The instrument type in Andromeda searches was set to Orbitrap and the precursor mass tolerance was set to ±20 ppm (first search) and ±4.5 ppm (main search). The MS/MS match tolerance was set to ±0.5 Da. The peptide spectrum match FDR and the protein FDR were set to 0.01 (based on target-decoy approach). The minimum peptide length analysed was 7 amino acids. For protein quantification unique and razor peptides were allowed. Modified peptides were allowed for quantification. The minimum score for modified peptides was 40. Retention times were recalibrated based on the built-in nonlinear time-rescaling algorithm. MS/MS identifications were transferred between LC-MS/MS runs with the “match between runs” option in which the maximal match time window was set to 0.7 min and the alignment time window set to 20 min. The quantification is based on the “value at maximum” of the extracted ion current. At least two quantitation events were required for a quantifiable protein. Further analysis and filtering of the results was done in Perseus v 1.6.8.0. (Tyanova et al. 2016). For quantification, we combined related biological replicates to categorical groups and investigated only those proteins that were found in at least one categorical group in a minimum of 3 out of 3 biological replicates.

References

Cox J, Neuhauser N, Michalski A, Scheltema RA, Olsen JV, Mann M. (2011) Andromeda: a peptide search engine integrated into the MaxQuant environment. *J Proteome Res.* **10**:1794-805.

Cox J, Mann M. (2008) MaxQuant enables high peptide identification rates, individualized p.p.b.-range mass accuracies and proteome-wide protein quantification. *Nat Biotechnol.* **26**:1367-72.

Cox J, Hein MY, Luber CA, Paron I, Nagaraj N, Mann M. (2014) Accurate proteome-wide label-free quantification by delayed normalization and maximal peptide ratio extraction, termed MaxLFQ. *Mol Cell Proteomics.* **13**:2513-26.

Kourelis J, Kaschani F, Grosse-Holz FM, Homma F, Kaiser M, van der Hoorn RAL. (2019) A homology-guided, genome-based proteome for improved proteomics in the alloploid *Nicotiana benthamiana*. *BMC Genomics.* **20**:722.

Qui WW, Xu J, Li X, Zhong L, Li JY, Li J, Nan FJ. (2009) Design and synthesis of matrix metalloprotease photoaffinity trimodular probes. *Chinese J. Chem.* **27**:825-833.

Shevchenko A, Tomas H, Havlis J, Olsen JV, Mann M. (2006) In-gel digestion for mass spectrometric characterization of proteins and proteomes. *Nat Protoc.* **1**:2856-2860.

Tyanova S, Temu T, Sinitcyn P, Carlson A, Hein MY, Geiger T, Mann M, Cox J. (2016) The Perseus computational platform for comprehensive analysis of (prote)omics data. *Nat Methods.* **13**:731-40.

Weerapana E, Speers AE, Cravatt BF. (2007) Tandem orthogonal proteolysis-activity-based protein profiling (TOP-ABPP)-a general method for mapping sites of probe modification in proteomes. *Nat Protoc.* **2**:1414-25.
