# Supplementary material for: Broad‐range metalloprotease profiling in plants uncovers immunity provided by defence‐related metalloenzyme
Source: New Phytol. 2022 May 26;235(3):1287–301. doi: 10.1111/nph.18200 (PMC9322406; doi:10.1111/nph.18200)
Supplement: Supplementary file 5 — Table S1 Plasmids. Table S2 Oligonucleotides. Please note: Wiley Blackwell are not responsible for the content or functionality of any Supporting Information supplied by the authors. Any queries (other than missing material) should be directed to the New Phytologist Central Office. [file NPH-235-1287-s001.pdf]

New Phytologist Supporting Information

Broad-range metalloprotease profiling in plants uncovers immunity provided by defence-related metalloenzyme

Kyoko Morimoto, Daniel Krahn, Farnusch Kaschani, Digby Hopkinson-Woolley, Anna Gee, Pierre Buscaill, Shabaz Mohammed, Stephan A. Sieber, Benjamin F. Cravatt, Christopher J. Schofield, Renier A. L. van der Hoorn

Article acceptance date 14 April 2022

Supplemental **Table S1** Plasmids used in our work.

**Plasmid**	**Description**	**Reference**
pICH51288	pL0M-PU-35S-TMV-3-51288	Engler *et al.* (2014)
pICH41414	pL0M-T-35S-1-41414	Engler *et al.* (2014)
pKM005	His-PRp27	this study
pKM008	His-PRp27 H122F	this study
pKM011	His-PRp27 E123Q	this study
pKM015	His-PRp27 H126F	this study
pJP001	pL0M-PU-35S-TMV-3-51288	Engler *et al.* (2014)
pJK001c	pL1V2-F1	Paulus *et al.* (2020)
pJP002	pL0M-T-35S-1-41414	Engler *et al.* (2014)
pJK037	pL2M-TRV2	this study
pJK082	pL1VE-R (pET28b)	Kourelis *et al.* (2020)
pJK122i	pL0M-S-His6-TEV	Kourelis *et al.* (2020)
pPB046	*35S:PRp27*	this study
pKM001	*35S:PRp27E123Q*	this study
pKM002	*35S:PRp27H122F*	this study
pKM003	*35S:PRp27H126F*	this study
pPB037	*TRV2::PRp27*	this study

References

Engler C, Youles M, Gruetzner R, Ehnert TM, Werner S, Jones JD, Patron NJ, Marillonnet S. 2014. A golden gate modular cloning toolbox for plants. *ACS Synth Biol.* **3:** 839-43.

Kourelis J, Malik S, Mattinson O, Krauter S, Kahlon PS, Paulus JK, Van der Hoorn RAL. 2020. Evolution of a guarded decoy protease and its receptor in solanaceous plants. *Nat. Commun.* **11:** 4393.

Paulus JK, Kourelis J, Ramasubramanian S, Homma F, Godson A, Hörger AC, Hong TN, Krahn D, Ossorio Carballo L, Wang S, et al. 2020. Extracellular proteolytic cascade in tomato activates immune protease Rcr3. *Proc. Nat. Acad. Sci. USA* **117:** 17409-17417.

Supplemental **Table S2** oligonucleotides.

**no**	**Description**	**Sequence (5´-3´) (*Bsa*I sites underlined, ligation sites in bold)**

1	NbPRp27-Fwd	tggtggtctca**aATG**GCTCATCACAAGATTTTCTT
2	NbPRp27-Rev	ttcgtggtctca**aagc**CTATGCGCGAAATTTGGC
3	NbPRp27-VIGS-Fwd	ttggtctca**attc**ATGGCTCATCACAAGATTTTC
4	NbPRp27-VIGS-Rev	ttggtctca**atcc**AACATGAATCTCATTGTTGC
5	NbPR27_pJK122i_For	TTGGTCTCAAGGTGCAGTTGATTACTCTGTGG
6	NbPR27_pJK122i_Rev	TTGGTCTCAAAGCCTATGCGCGAAATTTGGCTTTATAATC
7	NbPR27-H122F_Rev	TTCGTGGTCTCATGCTCTCGAAGTACAATACTCC
8	NbPR27-E123Q_For	TGGTGGTCTCAAGCACCCACGTT TGGCA
9	NbPR27-E123Q_Rev	TTCGTGGTCTCATGCTCTGGTGGTACAATACTCC
10	NbPR27-H126F_For	TGGTGGTCTCAAGCACCTTCGTTTGGCAGT
11	NbPR27-H126F_Rev	TTCGTGGTCTCATGCTCTCGTGGTACAATACTCC

Sequence A: PRp27 silencing fragment (from cDNA)

ttggtctcaattcATGGCTCATCACAAGATTTTCTTCATTTCTTCTCTATTTTTCCTAGCAATGTTC
ACCCAAAAAATCCATGCAGTTGATTACTCTGTGGCCAACACGGCCACAAACACCGCCGG
CGGTGCCCGTTTCAACCGAGATATCGGTGCTCAATACAGCCAGCAAACACTGGAAGCTG
CTACTTCATTCATATGGAATACCTTCCAGCAGAATTCTCCAGCTGACCGCAAAAATGTGC
AAAAGGTAAGCATGTTCGTTGACGACATGGACGGAGTAGCTTACGCTAGCAACAATGAG
ATTCATGTTggattgagaccaa

Sequence B: PRp27 ORF (from cDNA)

tggtggtctcaaATGGCTCATCACAAGATTTTCTTCATTTCTTCTCTATTTTTCCTAGCAATGTTC
ACCCAAAAAATCCATGCAGTTGATTACTCTGTGGCCAACACGGCCACAAACACCGCCGG
CGGTGCCCGTTTCAACCGAGATATCGGTGCTCAATACAGCCAGCAAACACTGGAAGCTG
CTACTTCATTCATATGGAATACCTTCCAGCAGAATTCTCCAGCTGACCGCAAAAATGTGC
AAAAGGTAAGCATGTTCGTTGACGACATGGACGGAGTAGCTTACGCTAGCAACAATGAG
ATTCATGTTAGTGCCAGGTACATCCAAGGTTACTCCGGTGACGTCAGGAGAGAGATTACT
GGAGTATTGTACCACGAGAGCACCCACGTTTGGCAGTGGAATGGGAATGGTGGGGCTCC
AGGCGGTTTAATTGAAGGGATTGCTGATTATGTGAGGCTCAAAGCCGGTTTCGGACCTAG
CCACTGGGTGAAACCAGGCCAGGGCGACCGATGGGACCAGGGCTATGACGTGACTGCTC
GATTTCTTGATTATTGCAACAGCTTGAGAAATGGGTTCGTGGCACAACTTAACAAAAAGA
TGAGAACTGGCTATAGTAATCAGTTCTTTGTTGACTTGTTGGGGAAGACGGTTGATCAAC
TTTGGAATGATTATAAAGCCAAATTTCGCGCATAG (SP-encoding sequence underlined)
